# Supplementary material for: Improving risk assessments in conservation ecology
Source: Nat Commun. 2019 Jun 27;10:2836. doi: 10.1038/s41467-019-10700-4 (PMC6597725; doi:10.1038/s41467-019-10700-4)
Supplement: Supplementary file 1 — Supplementary Information [file 41467_2019_10700_MOESM1_ESM.pdf]

Supplementary information

Improving risk assessments in conservation ecology

Kotaro Ono, Øystein Langangen, Nils Chr. Stenseth

Contents

Supplementary Figure 1 2
Supplementary Figure 2 3
Supplementary Figure 3 4
Supplementary Figure 4 5
Supplementary Figure 5 6
Supplementary Figure 6 7
Supplementary Figure 7 8
Supplementary Figure 8 9
Supplementary Table 1 10
References 10

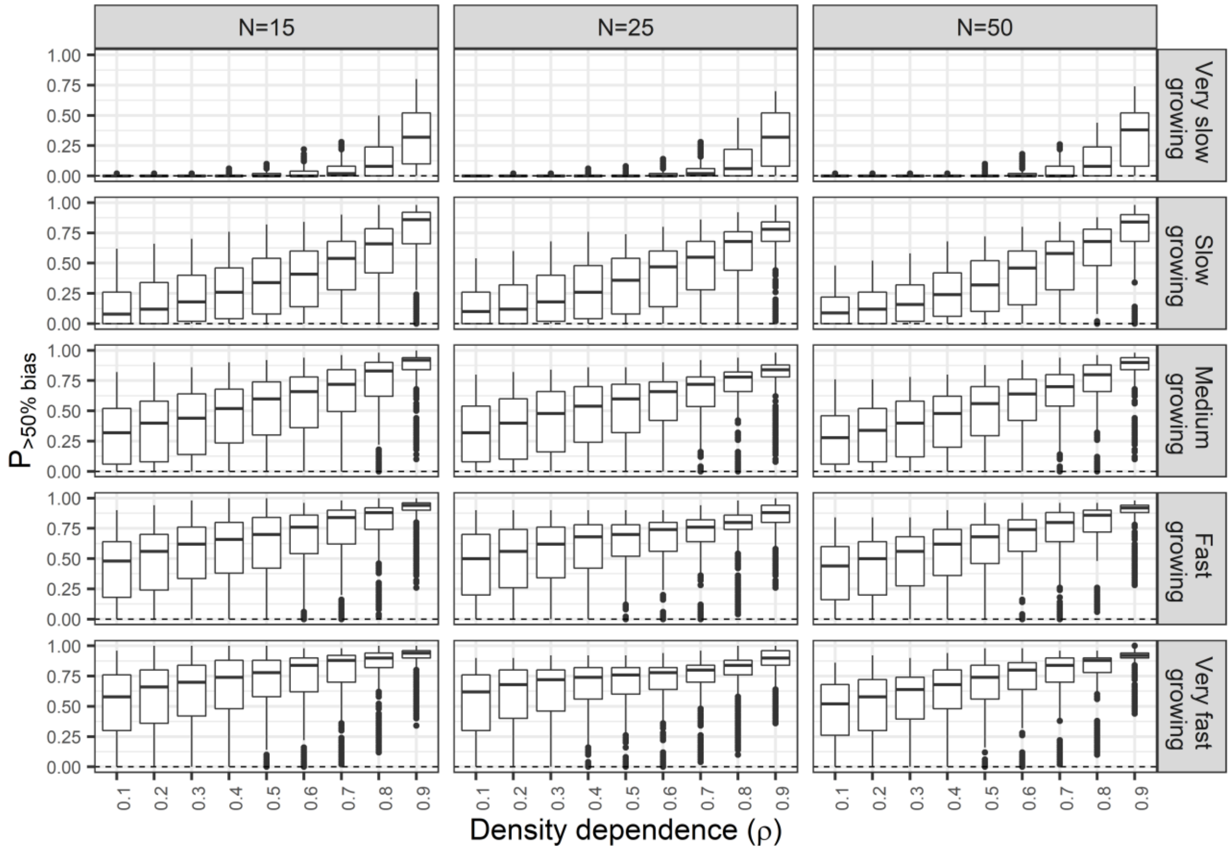

Supplementary Figure 1

Probability that the estimate of final year depletion is at least 50% biased (absolute relative error rate >0.5) as a function of time series length (columns), population growth potential (rows), and density dependence (x-axis in each panel). Growth potential is divided into categories based on the species intrinsic rate of growth from very slow-growing (0-0.3), slow-growing (0-3-0.7), medium-growing (0.7-1.1), fast-growing (1.1-1.5), to very fast-growing (>1.5) species. The center line in the boxplot is the median, box limits represent the first and third quartile, whiskers show the 1.5 x interquartile range, and the points are the outliers.

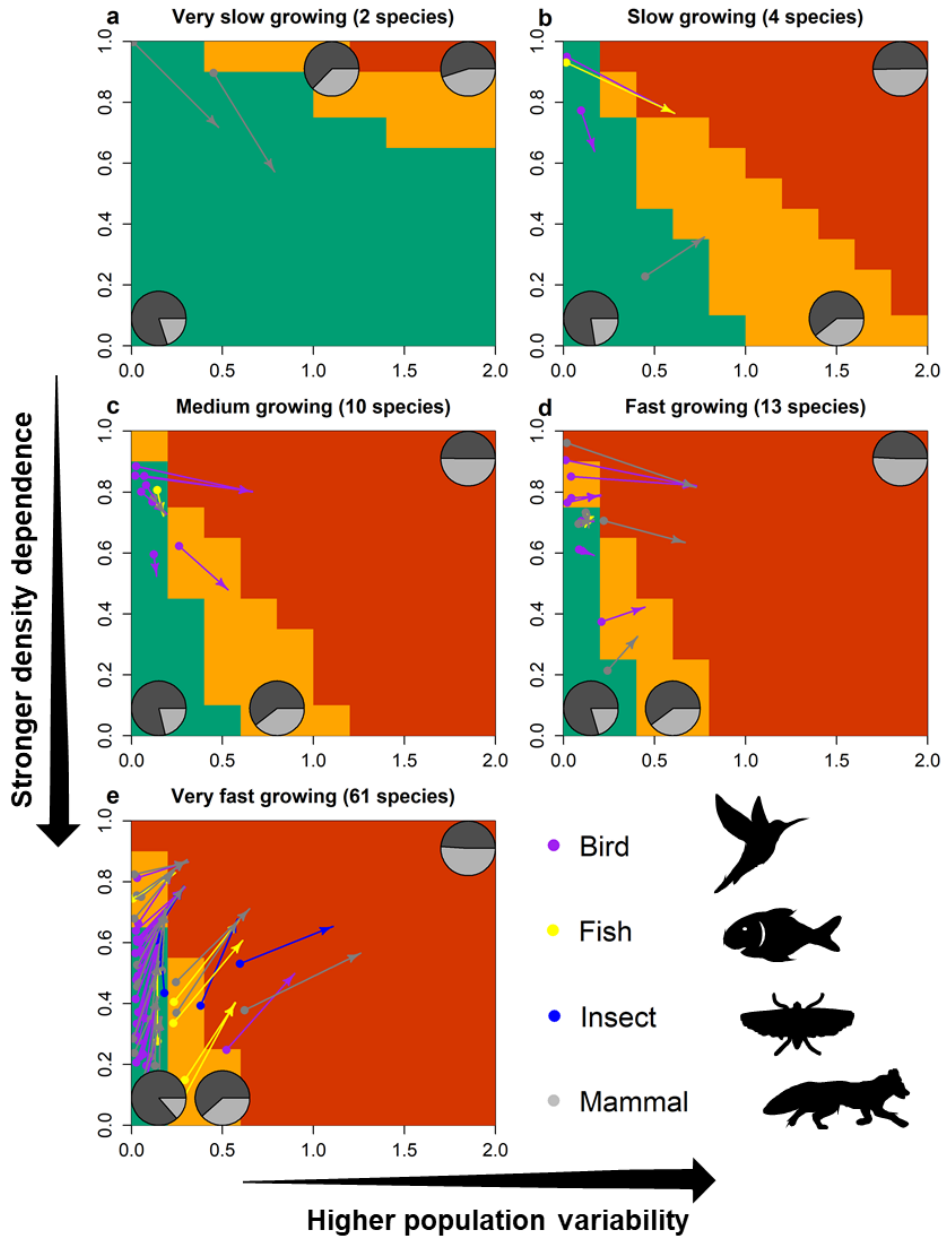

Supplementary Figure 2

Risk maps of biased population assessment across four taxonomic groups (birds, fish, insects, and mammals) with a time series length ranging from 11 to 20 years. Risks are quantified as having more than 60% (orange-red), 30 to 60% (orange), and below 30% (bluish-green) chance of estimating the final year depletion level (how much the population has changed compared to the start of the time series) with at least 50% bias in both directions. Risk are quantified based on the results from extensive (135,000 scenarios) simulation-estimation study. The risk map is summarized in 2D based on total (both process and observation) population variability measured in terms of coefficient of variation and population density dependence. The plot is restricted to the parameter range used in the simulation studies. The panels are organized by the species intrinsic rate of growth from very slow-growing (0-0.3) (**a**), slow-growing (0-3-0.7) (**b**), medium-growing (0.7-1.1) (**c**), fast-growing (1.1-1.5) (**d**), to very fast-growing (>1.5)) (**e**) species. The filled dots are the estimated parameter values for the four taxonomic groups (birds (purple), fish (grey), insects (blue), and mammals (yellow)) and the arrows show the most plausible (“true”) parameter values that could have generated such estimates. The pie chart within each panel identifies the sign of estimation bias for each risk category i.e. false-positive in dark grey and false-negative in light grey.

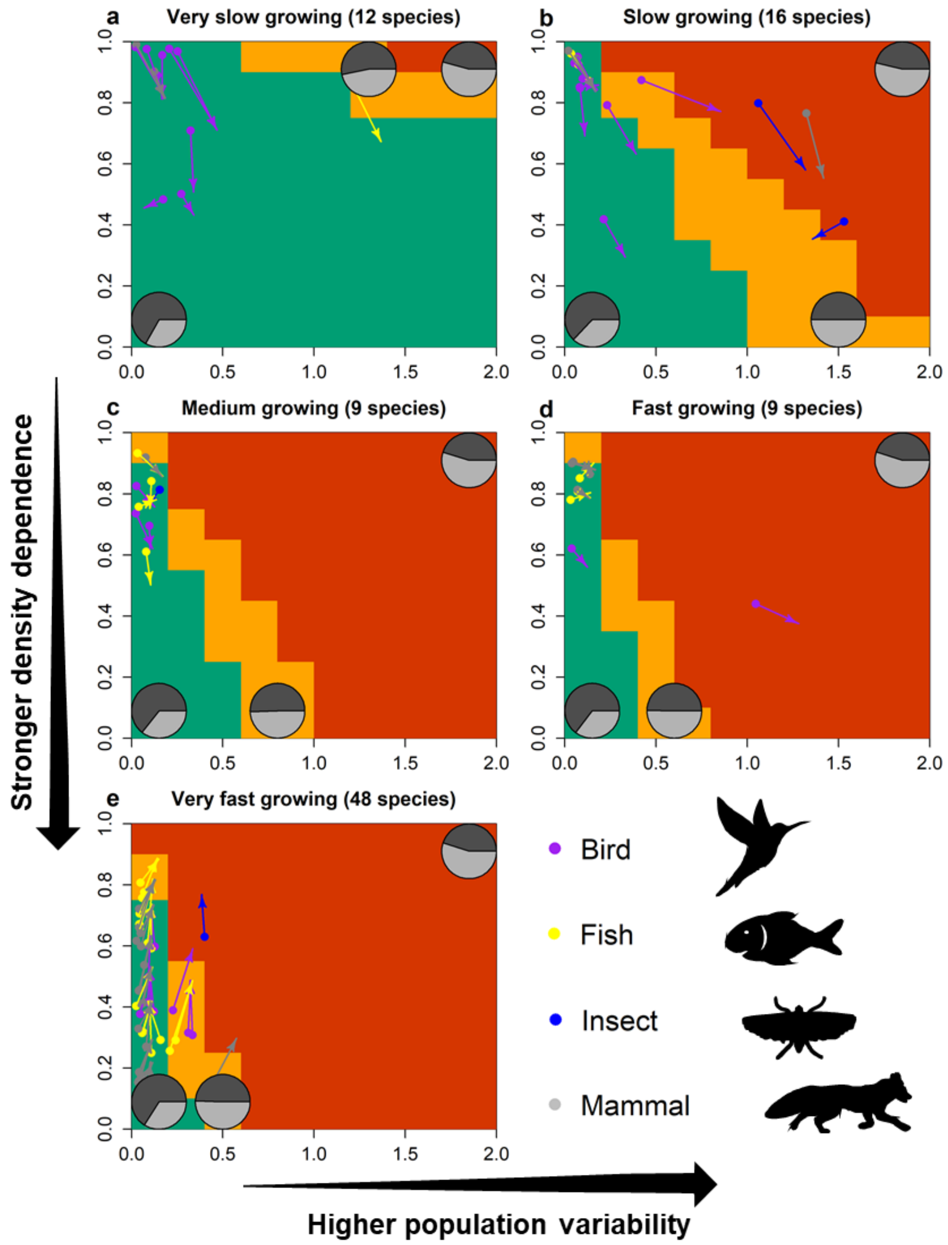

Supplementary Figure 3

Risk maps of biased population assessment across four taxonomic groups (birds, fish, insects, and mammals) with a time series length ranging from 31 to 65 years. Risks are quantified as having more than 60% (orange-red), 30 to 60% (orange), and below 30% (bluish-green) chance of estimating the final year depletion level (how much the population has changed compared to the start of the time series) with at least 50% bias in both directions. Risk are quantified based on the results from extensive (135,000 scenarios) simulation-estimation study. The risk map is summarized in 2D based on total (both process and observation) population variability measured in terms of coefficient of variation and population density dependence. The plot is restricted to the parameter range used in the simulation studies. The panels are organized by the species intrinsic rate of growth from very slow-growing (0-0.3) (**a**), slow-growing (0-3-0.7) (**b**), medium-growing (0.7-1.1) (**c**), fast-growing (1.1-1.5) (**d**), to very fast-growing (>1.5)) (**e**) species. The filled dots are the estimated parameter values for the four taxonomic groups (birds (purple), fish (grey), insects (blue), and mammals (yellow)) and the arrows show the most plausible (“true”) parameter values that could have generated such estimates. The pie chart within each panel identifies the sign of estimation bias for each risk category i.e. false-positive in dark grey and false-negative in light grey.

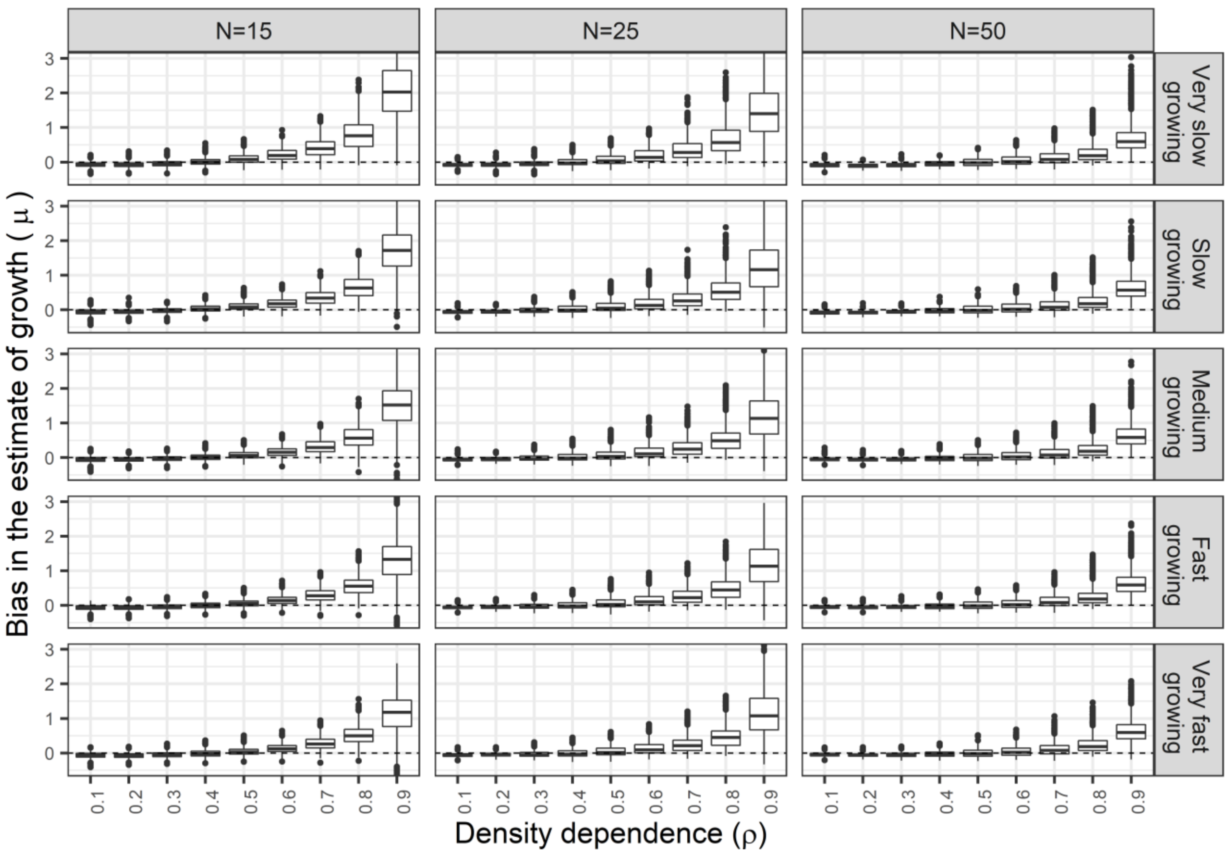

Supplementary Figure 4

Bias (calculated as the median relative error between the estimate and the true value) in the estimate of growth potential as a function of time series length (columns), population growth potential (rows), and density dependence (x-axis in each panel). Growth potential is divided into categories based on the species intrinsic rate of growth from very slow-growing (0-0.3), slow-growing (0-3-0.7), medium-growing (0.7-1.1)**,** fast-growing (1.1-1.5), to very fast-growing (>1.5) species. The center line in the boxplot is the median, box limits represent the first and third quartile, whiskers show the 1.5 x interquartile range, and the points are the outliers.

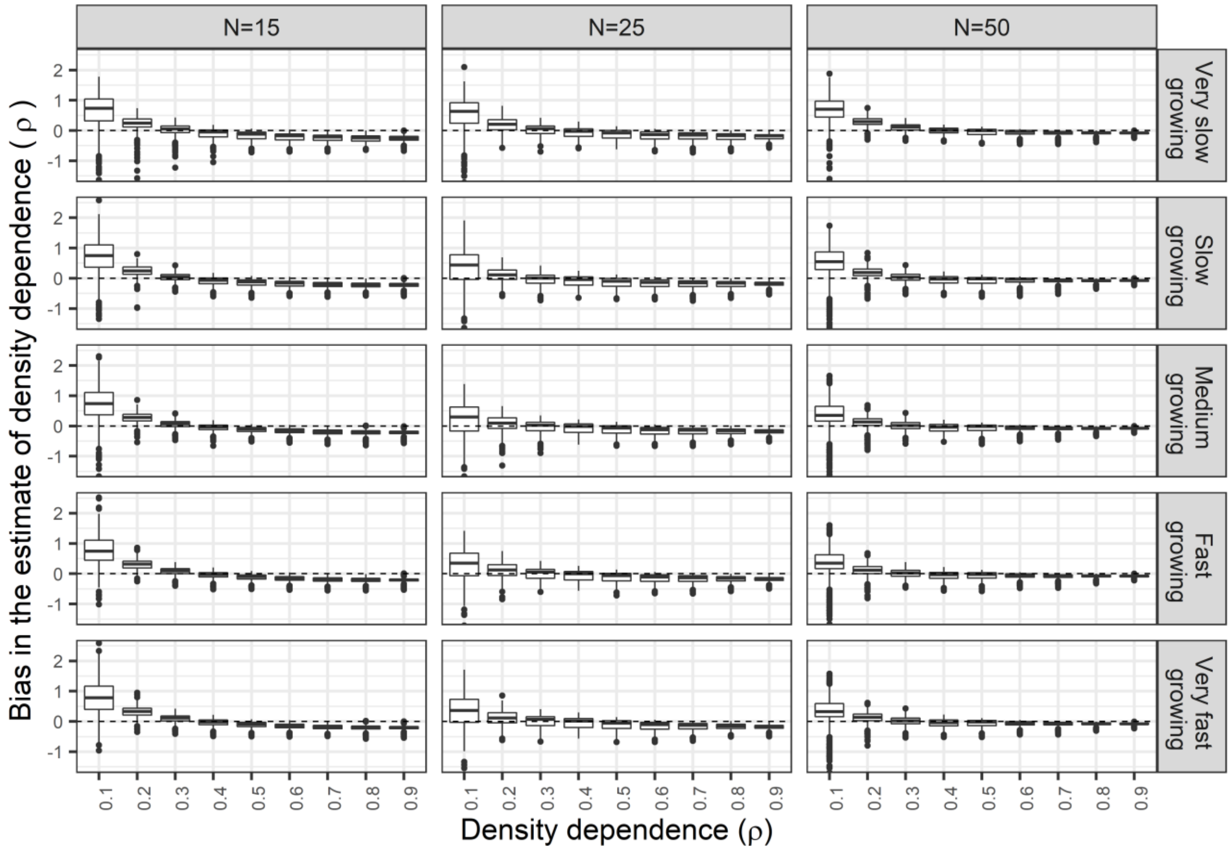

Supplementary Figure 5

Bias (calculated as the median relative error between the estimate and the true value) in the estimate of density dependence as a function of time series length (columns), population growth potential (rows. Divided into categories based on the species intrinsic rate of growth from very slow-growing (0-0.3), slow-growing (0-3-0.7), medium-growing (0.7-1.1)**,** fast-growing (1.1-1.5), to very fast-growing (>1.5) species), and density dependence (x-axis in each panel). The center line in the boxplot is the median, box limits represent the first and third quartile, whiskers show the 1.5 x interquartile range, and the points are the outliers.

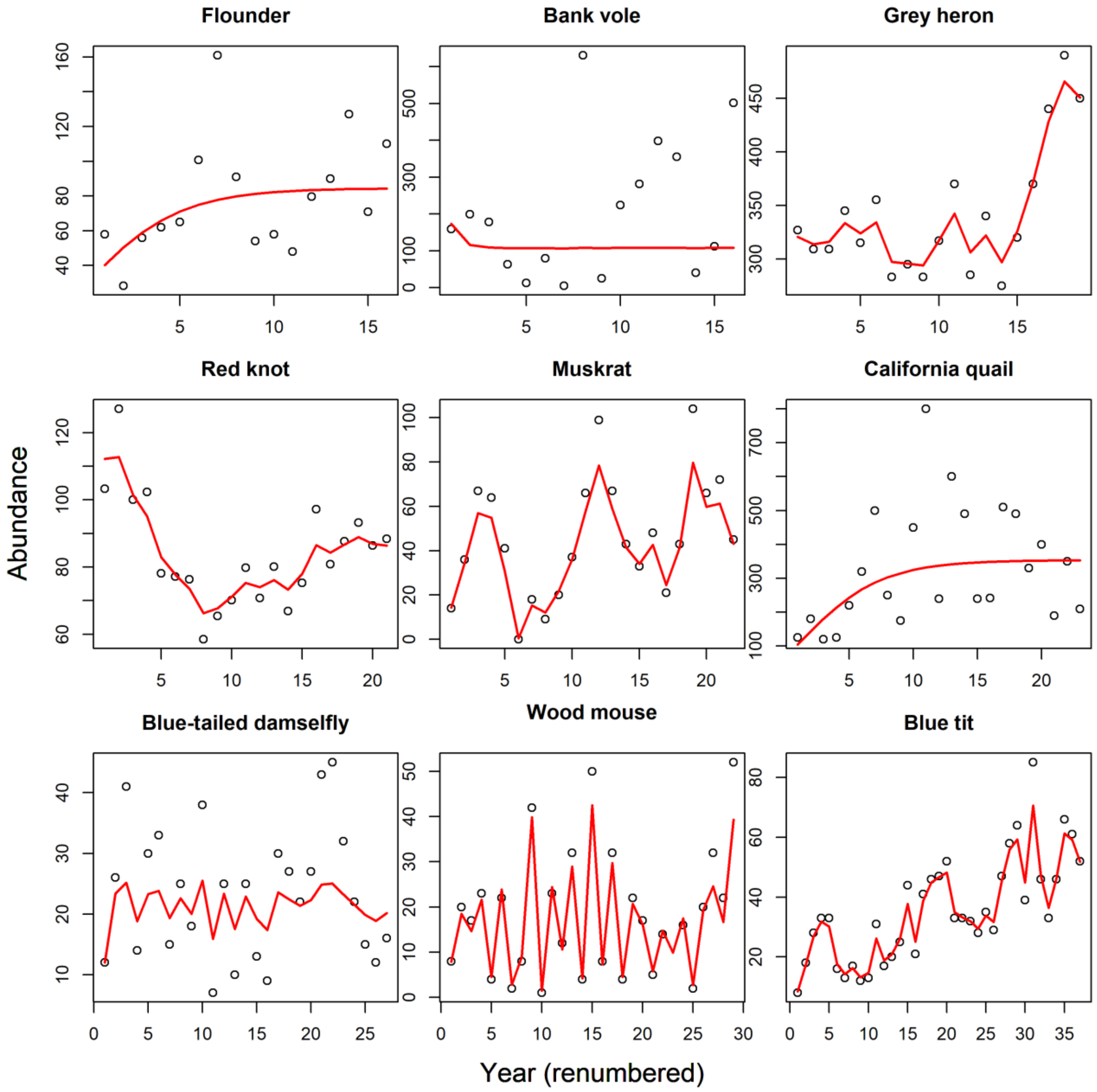

Supplementary Figure 6

Selected examples of population time series (black dot) from the GPDD dataset along with the predictions based on GSSMs fit to the data (red line).

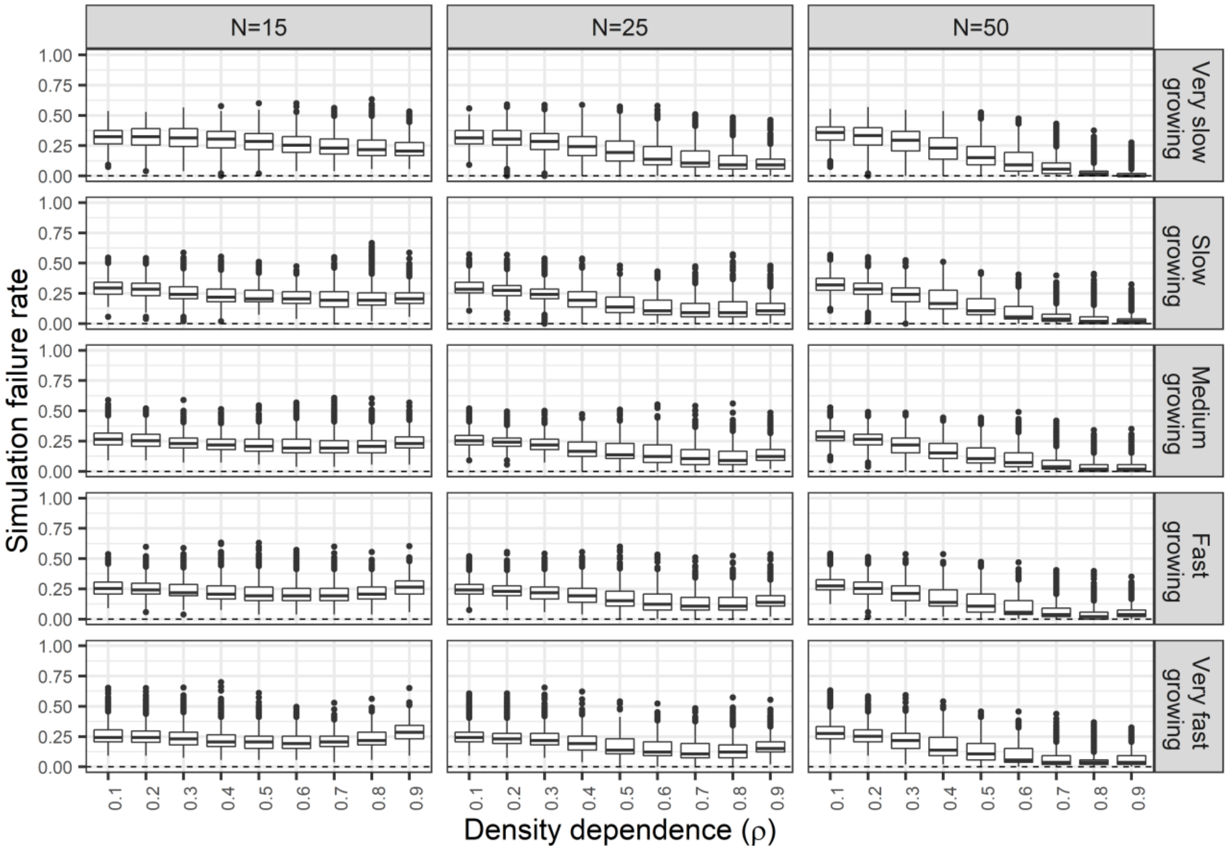

Supplementary Figure 7

Simulation failure rate (i.e. the probability that the GSSM does not converge when fitted to dataset generated from a scenario) as a function of time series length (columns), population growth potential (rows), and density dependence (x-axis in each panel). Growth potential is divided into categories based on the species intrinsic rate of growth from very slow-growing (0-0.3), slow-growing (0-3-0.7), medium-growing (0.7-1.1)**,** fast-growing (1.1-1.5), to very fast-growing (>1.5) species. The center line in the boxplot is the median, box limits represent the first and third quartile, whiskers show the 1.5 x interquartile range, and the points are the outliers.

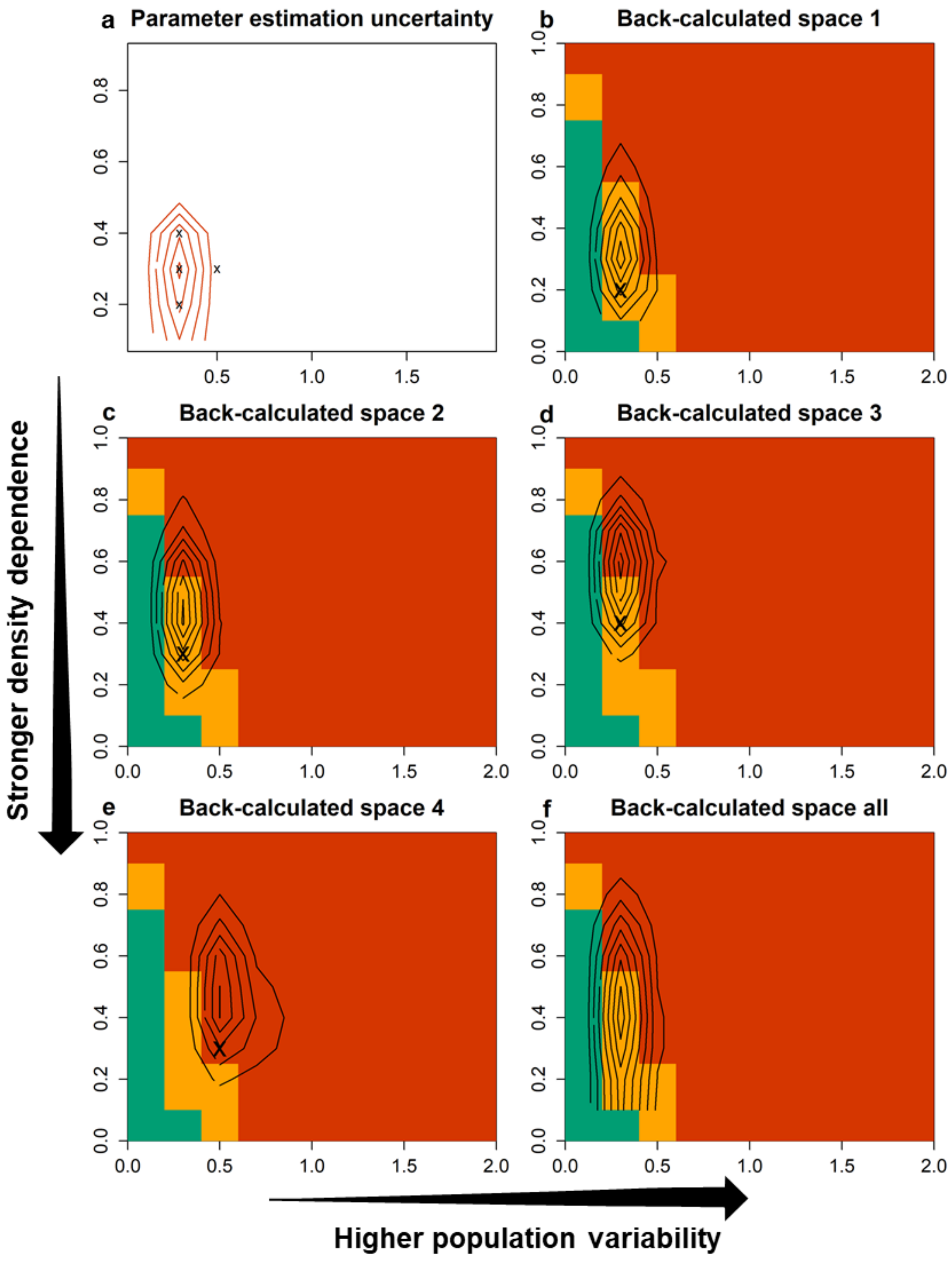

Supplementary Figure 8

Illustrative example of the influence of parameter estimation uncertainty on the back-calculated true parameter space. Parameter estimation uncertainty around the estimate of total population variability (CV) and density dependence from a bird (indigo blunting) population observation time series of 50 years. The 4 crosses in the figure show exemplary points for which true parameter space is back-calculated (**a**). Back-calculated true parameter space for each exemplary point estimates (**b-e**). The final back-calculated true parameter space that takes into account parameter estimation uncertainty (i.e. a weighted average of all back-calculated parameter space for each of the 3000-bootstrap samples. Bootstrap samples are obtained from a multivariate normal distribution centered around the parameters maximum likelihood estimates with the corresponding covariance matrix).

Supplementary Table 1

Parameter definition, values used for scenarios, and the corresponding categorization for estimated parameters

**Parameter**	**Simulation values**	**Binning for estimated values**	**Reference**
μ (productivity)	0.1, 0.5, 0.9, 1.3, 1.7	[0,0.3), [0.3,0.7), [0.7,1.1), [1.1,1.5), [1.5,100)	1
ρ (level of density dependence)	0.1, 0.2, 0.3, 0.4, 0.5, 0.6, 0.7, 0.8, 0.9	[0,0.15), [0.15,0.25), [0.25,0.35), [0.35,0.45), [0.45,0.55), [0.55,0.65), [0.65,0.75), [0.75,0.85), [0.85,1)	1–3
Initial depletion	0.1, 0.3, 0.5, 0.7, 0.9, 1.1, 1.3, 1.5, 1.7, 1.9	[0,0.2), [0.2,0.4), [0.4,0.6), [0.6,0.8), [0.8,1.0), [1.0,1.2), [1.2,1.4), [1.4,1.6), [1.6,1.8), [1.8,10)	4
Time series length (years)	15, 25, 50	[11,20], [21,30], [31,65]	1,5
Population variability (CV around equilibrium population)	0.1, 0.3, 0.5, 0.7, 0.9, 1.1, 1.3, 1.5, 1.7, 1.9	[0,0.2), [0.2,0.4), [0.4,0.6), [0.6,0.8), [0.8,1.0), [1.0,1.2), [1.2,1.4), [1.4,1.6), [1.6,1.8), [1.8,2)	2,6
Ratio observation to process error variance	0.1, 0.2, 0.3, 0.4, 0.5, 0.75, 1.0, 2.0, 4.5, 9	[0,0.15), [0.15,0.25), [0.25,0.35), [0.35,0.45), [0.45,0.625), [0.625,0.875), [0.875,1.5), [1.5,3.25), [3.25,6.75), [6.75,20)	2,7

References

1. Fagan, W. F., Meir, E. & Moore, J. L. Variation thesholds for extinction and their implications for conservation strategies. *Am. Nat.* **154,** 510–520. (1999).
2. Bunnefeld, N., Hoshino, E. & Milner-Gulland, E. J. Management strategy evaluation: A powerful tool for conservation? *Trends Ecol. Evol.* **26,** 441–447 (2011).
3. Keddy, P. A. *Wetland ecology: principles and conservation*. (2010).
4. Punt, A. E. Extending production models to include process error in the population dynamics. *Can. J. Fish. Aquat. Sci.* **60,** 1217–1228 (2003).
5. Knape, J. & de Valpine, P. Are patterns of density dependence in the Global Population Dynamics Database driven by uncertainty about population abundance? *Ecol. Lett.* **15,** 17–23 (2012).
6. Inchausti, P. & Halley, J. On the relation between temporal variability and persistence time in animal populations. *J. Anim. Ecol.* **72,** 899–908 (2003).
7. Connors, B. M., Cooper, A. B., Peterman, R. M. & Dulvy, N. K. The false classification of extinction risk in noisy environments. *Proc. R. Soc. B Biol. Sci.* **282,** (2015).
