# Supplementary material for: Improving risk assessments in conservation ecology
Source: Nat Commun. 2019 Jun 27;10:2836. doi: 10.1038/s41467-019-10700-4 (PMC6597725; doi:10.1038/s41467-019-10700-4)
Supplement: Supplementary file 3 — Reporting Summary [file 41467_2019_10700_MOESM3_ESM.pdf]

nature research

Corresponding author(s): Kotaro Ono, Øystein Langangen, Nils Chr. Stenseth

Last updated by author(s): May 6, 2019

Reporting Summary

Nature Research wishes to improve the reproducibility of the work that we publish. This form provides structure for consistency and transparency in reporting. For further information on Nature Research policies, see Authors & Referees and the Editorial Policy Checklist.

Statistics

For all statistical analyses, confirm that the following items are present in the figure legend, table legend, main text, or Methods section.

n/a	Confirmed	
☐	☒	The exact sample size (*n*) for each experimental group/condition, given as a discrete number and unit of measurement
☒	☐	A statement on whether measurements were taken from distinct samples or whether the same sample was measured repeatedly
☒	☐	The statistical test(s) used AND whether they are one- or two-sided<br>*Only common tests should be described solely by name; describe more complex techniques in the Methods section.*
☒	☐	A description of all covariates tested
☒	☐	A description of any assumptions or corrections, such as tests of normality and adjustment for multiple comparisons
☐	☒	A full description of the statistical parameters including central tendency (e.g. means) or other basic estimates (e.g. regression coefficient) AND variation (e.g. standard deviation) or associated estimates of uncertainty (e.g. confidence intervals)
☒	☐	For null hypothesis testing, the test statistic (e.g. *F*, *t*, *r*) with confidence intervals, effect sizes, degrees of freedom and *P* value noted<br>*Give P values as exact values whenever suitable.*
☒	☐	For Bayesian analysis, information on the choice of priors and Markov chain Monte Carlo settings
☒	☐	For hierarchical and complex designs, identification of the appropriate level for tests and full reporting of outcomes
☒	☐	Estimates of effect sizes (e.g. Cohen's *d*, Pearson's *r*), indicating how they were calculated

Our web collection on statistics for biologists contains articles on many of the points above.

Software and code

Policy information about availability of computer code

Data collection	All data supporting the findings of this study are made available in the supplementary information files. GPDD data can also be downloaded from: https://www.imperial.ac.uk/cpb/gpdd2/secure/login.aspx
Data analysis	All analysis were performed using R (version 3.5.2) and the code and packages used in the analysis are all made available in the supplementary information files

For manuscripts utilizing custom algorithms or software that are central to the research but not yet described in published literature, software must be made available to editors/reviewers. We strongly encourage code deposition in a community repository (e.g. GitHub). See the Nature Research guidelines for submitting code & software for further information.

Data

Policy information about availability of data

All manuscripts must include a data availability statement. This statement should provide the following information, where applicable:

- Accession codes, unique identifiers, or web links for publicly available datasets
- A list of figures that have associated raw data
- A description of any restrictions on data availability

The authors declare that all data supporting the findings of this study are available within the paper, its supplementary information files, and from https://www.imperial.ac.uk/cpb/gpdd2/secure/login.aspx

Field-specific reporting

Please select the one below that is the best fit for your research. If you are not sure, read the appropriate sections before making your selection.

☐ Life sciences ☐ Behavioural & social sciences ☒ Ecological, evolutionary & environmental sciences

For a reference copy of the document with all sections, see nature.com/documents/nr-reporting-summary-flat.pdf

Ecological, evolutionary & environmental sciences study design

All studies must disclose on these points even when the disclosure is negative.

Study description	Using the GPDD database of species (different taxa) abundance time series along with a simulation-estimation procedure, we show in this study how management-relevant population parameter could be biased and how we can potentially correct for it
Research sample	generated from simulation and the time series of species abundance is available from https://www.imperial.ac.uk/cpb/gpdd2/secure/login.aspx
Sampling strategy	Not all data sets in the GPDD are reliable. Thus, data were filtered out from the database using the same criteria as in Knape and de Valpine (2012)
Data collection	generated from simulation and available from https://www.imperial.ac.uk/cpb/gpdd2/secure/login.aspx
Timing and spatial scale	see https://www.imperial.ac.uk/cpb/gpdd2/secure/login.aspx for more information on each dataset
Data exclusions	Not all data sets in the GPDD are reliable. Thus, data were filtered out from the database using the same criteria as in Knape and de Valpine (2012) i.e. harvest and non-index based data were removed, as well as data sampled at non-annual intervals and time series with less than 15 unique values
Reproducibility	We provided the full code and data to reproduce the study
Randomization	not applicable because the study uses simulation-estimation to correct for inherent bias present in a model fit to data
Blinding	not applicable in this study as we use existing field collected data

Did the study involve field work? ☐ Yes ☒ No

Reporting for specific materials, systems and methods

We require information from authors about some types of materials, experimental systems and methods used in many studies. Here, indicate whether each material, system or method listed is relevant to your study. If you are not sure if a list item applies to your research, read the appropriate section before selecting a response.

Materials & experimental systems

n/a	Involved in the study	
☒	☐	Antibodies
☒	☐	Eukaryotic cell lines
☒	☐	Palaeontology
☒	☐	Animals and other organisms
☒	☐	Human research participants
☒	☐	Clinical data

Methods

n/a	Involved in the study	
☒	☐	ChIP-seq
☒	☐	Flow cytometry
☒	☐	MRI-based neuroimaging
